# The Effects of Different Calcium Channel Blockers on Aldosterone-Producing Adenoma Cells

**DOI:** 10.3389/fendo.2020.00260

**Published:** 2020-04-28

**Authors:** Fen Wang, Xiaosen Ma, Anli Tong, Yushi Zhang, Jin Wen, Yuxiu Li

**Affiliations:** ^1^Department of Endocrinology, Key Laboratory of Endocrinology, National Health Commission of the People's Republic of China, Peking Union Medical College Hospital, Peking Union Medical College, Chinese Academy of Medical Sciences, Beijing, China; ^2^Department of Endocrinology, Tongji Hospital, Tongji Medical College, Huazhong University of Science and Technology, Wuhan, China; ^3^Department of Urology, Peking Union Medical College Hospital, Peking Union Medical College, Chinese Academy of Medical Sciences, Beijing, China

**Keywords:** aldosterone-producing adenoma, calcium channel blocker, ACTH, angiotensin II, benidipine

## Abstract

**Purpose:** The aim of this study is to examine the effects of different kinds of calcium channel blockers (CCBs) on primary aldosterone-producing adenoma (APA) mainly with *KCNJ5* mutations. Primary cultured APA cells were treated with different calcium channel blockers (L/T type CCB benidipine, T-type CCB mibefradil and L-type CCB nifedipine), and aldosterone secretagogues with or without nifedipine. Aldosterone level, aldosterone synthase (CYP11B2) mRNA expression and cell proliferation were detected. The results showed that all three CCBs significantly inhibit aldosterone secretion and CYP11B2 mRNA expression. Benidipine was relatively more effective than mibefradil or nifedipine. In addition, only mibefradil marginally inhibited cell proliferation. Adrenocorticotropin (ACTH) had a much stronger effect in stimulating aldosterone secretion and promoting cell proliferation from APA's than angiotensin II (ATII). Different from ACTH and ATII, potassium had no effect. Nifedipine inhibited the basal and ACTH-, ATII-elicited aldosterone secretion. Twenty three of 24 APAs had somatic *KCNJ5* mutation. In conclusion, benidipine, mibefradil and nifedipine significantly inhibit aldosterone secretion in primary cultured APA cells.

## Introduction

Primary aldosteronism is the most common secondary hypertension, accounting for about 10% of hypertension patients (1, 2), and is caused principally by aldosterone-producing adenoma (APA) and bilateral adrenal hyperplasia. Over the past years, the etiology has been identified in more than 50% of APA cases (3–5), involving several somatic gene mutations such as *KCNJ5, ATP2B3, ATP1A1*, and *CACNA1D* genes, with somatic *KCNJ5* being the predominant mutation. A meta-analysis of 1636 APA patients revealed that the overall prevalence of somatic *KCNJ5* mutations was 43%, ranging from 35% in Europe, the United States and Australia to 63% in Asia (3–5). Germline mutations of *KCNJ5, CACNA1D*, and *CACNA1H* genes were also found in patients with familial hyperaldosteronism (6, 7). *KCNJ5* gene codes G protein-activated inward rectifier potassium channel 4 (GIRK4). *ATP1A1* gene codes Na^+^/K^+^-ATPase in the cell membrane (8). When *KCNJ5* gene (9) and *ATP1A1* (8) gene mutate, the increased intracellular Na^+^ concentration will promote cell membrane depolarization, leading to opening of voltage-gated Ca^2+^ channels (VGCCs) (7, 10). Mutations in *CACNA1D* and *CACNA1H*, affecting the Cav1.3 subunit of the L-type VGCC and Cav3.2 subunit of the T-type VGCC, respectively, impair channel activation and inactivation. Calcium influx via opened VGCC results in increased synthesis of steroidogenic enzymes and aldosterone.

Calcium channel blockers (CCBs) inhibit calcium influx and aldosterone secretion in cell lines transfected with mutants of *KCNJ5* genes (11). Nonetheless, their effects on primary cultured APA cells are largely unknown. Only one study reported that L-type CCB nifedipine inhibited aldosterone production in primary cultures from three APA tissues (12). However, the effect of L/T -type CCBs or T-type CCBs on APAs are poorly understood.

Adrenocorticotropin (ACTH), angiotensin II (ATII) and potassium are the main regulators of aldosterone secretion in normal adrenal zona glomerulosa (ZAG) cells. Their roles in APA cells remained largely unknown. In this paper, we examined the effects of CCBs on APA. We utilized the primary culture of human APA cells, which might bear somatic gene mutations, and, presumably, better mimic the environment of human APAs. The aim of the study was (1) to observe the effect of different kinds of CCBs especially benidipine on the aldosterone secretion and proliferation of primary cultured APA cells; (2) to investigate the effect of the three physiological stimuli (ACTH, ATII and potassium) on the aldosterone production and proliferation of APA cells, and the effects of stimuli combined with CCB on APA cells.

## Materials and Methods

### Reagents

DMEM/F12 was procured from Hyclone (GE Healthcare, Boston, USA). Fetal bovine serum (FBS) was purchased from Tianhang Biotechnology Company (Zhejiang, China). Collagenase type I was bought from Gibco-Life Technologies (NewYork, USA). Red blood cell lysis buffer was from Solarbio company (Beijing, China). ITS+1, T-type CCB mibefradil and potassium chloride obtained from Sigma-Aldrich (Missouri, USA). L/T-type CCB benidipine and L-type CCB nifedipine came from MCE (New Jersey, USA). ACTH and ATII were products of Abcam (Cambridge, Britain). CCK-8 Assay Kit was from Dojindo Laboratories (Kyushu, Japan). Trizol reagent was produced by Invitrogen (Thermo Fisher Scientific, Massachusetts, USA). QIAamp DNA Mini Kit was from QIAGEN (Duesseldorf, German) and Quick-DNA™ FFPE Kit from Zymo (California, USA). Aldosterone and rennin activity radioimmunoassay kit was manufactured by the North Institute of Biotechnology (Beijing, China). PrimeScript™ RT reagent Kit with gDNA Eraser and TB Green™ Premix Ex Taq™ II were bought from Takara (Shiga, Japan).

### Subjects

APA tissues from 24 patients were collected for *in vitro* studies. Clinical features of the 24 APA patients were presented in Table 1. All patients had hypertension and hypokalemia. The minimum potassium concentration of number 13 and 15 was unknown. The diagnostic criteria of primary aldosteronism were as follows: PAC in upright position was above 13 ng/dl; plasma renin activity in upright position was below 0.5 ng/ml/h; aldosterone to renin activity ratio (ARR) was above 30 ng/dl per ng/ml/h; PAC was not suppressed by captopril challenge test. Classification of primary aldosteronism was based on computed tomography, with APA showing unilateral adrenal mass and normal opposite adrenal. All APAs were resected and pathologically diagnosed with adrenocortical adenoma.

**Table 1:** Clinical and genetic features of APA patients in *in vitro* study.

**Number**	**sex**	**Age**	**Duration of hypertension (months)**	**Duration of hypokalemia (months)**	**BPmax (mmHg)**	**Kmin (mmol/l)**	**Cr (umol/l)**	**PRA, ng/ml/h**	**PAC, ng/dl**	**ACTH (pg/ml)**	**UFC (μg)**	**Diameter of APA (mm)**	**Amino acid changes of *KCNJ5* gene**
1	F	52	120	24	180/100	2.9	64	0.02	15.45	7.8	48.15	20	G151R
2	M	44	36	36	220/140	2.0	131	0.01	34.00	49.5	35.28	13.4	WT
3	M	41	24	1	NA	2.5	70	0.01	18.17	21.7	100.32	19	G151R
4	F	37	1	1	180/120	3.2	55	0.04	19.38	19	NA	13	L168R
5	M	30	24	3	210/130	2.5	84	a	a	59.5	25.74	15	L168R
6	M	48	48	24	NA	1.6	105	0.01	23.46	60	64.39	25	G151R
7	M	34	48	6	180/120	2.6	91	0.01	15.09	NA	NA	19	G151R
8	M	53	60	1	180/110	1.7	118	a	a	NA	NA	18	E145Q
9	M	37	24	0.5	184/119	3.2	62	0.01	13.98	7.9	77.50	22	G151R
10	M	40	48	2	NA	2.3	73	0.01	17.21	39.4	156.03	20	L168R
11	F	57	204	2	180/110	1.4	85	0.01	18.25	14	41.60	14	L168R
12	F	31	120	0.25	250/140	2.6	78	0.01	24.1	NA	NA	18	L168R
13	F	42	72	6	220/110		65	0.43	13.69	8.3	17.42	14.3	G151R
14	F	54	144	72	190/120	2.7	58	0.01	25.71	102	52.80	15	G151R
15	F	35	6	6	NA		56	0.01	31.00	37.7	NA	18	E145Q
16	F	37	144	5	210/150	3.2	49	0.01	13.25	9.07	50.00	NA	c.469-477delATCACAGAG
17	M	64	72	24	180/110	1.9	80	0.09	13.97	46.3	68.89	13	G151R
18	F	32	5	2	207/127	3.1	54	0.04	16.60	27.3	NA	18	L168R
19	M	55	180	6	160/105	2.1	81	0.01	22.73	24.4	70.31	17	G151R
20	M	66	132	96	200/100	2.5	105	0.01	24.65	26.6	51.33	16	L168R
21	F	51	240	96	200/120	1.8	114	0.01	21.02	20	63.00	22	L168R
22	M	69	240	1	180/110	2.7	100	0.01	37.94	24.6	58.50	22	G151R
23	M	45	48	6	170/110	2.0	83	0.01	14.76	NA	NA	20	L168R
24	M	37	60	36	160/115	2.2	90	0.01	26.66	27.7	57.20	20	G151R
mean ± SD		45.5 ± 11.4	60^*^(33, 135)	6^*^ (1.8, 24)	192 ± 22/119 ± 13	2.4 ± 0.5	81 ± 22	0.01^*^ (0.01, 0.01)	20.96 ± 6.94	25.6^*^(17.8, 41.1)	57.20^*^(48.15, 68.89)	17.9 ± 3.3	

BP max, the maximum blood pressure; K min: the minimal potassium concentration; Cr, creatine; UFC: urine free cortisol; PRA, plasma renin activity; PAC, plasma aldosterone concentration; NA, not available;

* Median (25%, 75%);

a *The values were detected in another hospital with a different method*.

### Primary Culture of APA Cells

The fresh APA tissues were digested in 2% collagenase type I for 2 h. Red blood cells were removed by red blood cell lysis buffer. Dispersed cells were harvested by centrifugation at 1,500 rpm for 5 min and washed once. The cells were plated into 24-well plates at a density of 5 × 10^4^ cells/well, or into 96-well plates at a density of 10^4^ cells/well. APA cells were cultured in DMEM/F12 medium supplemented with 1% ITS+1 and 10% FBS at 37°C in a 5% CO_2_ atmosphere. The following experiments were carried out on the 3 day of cell culture.

### Dose-Effect Studies of CCBs

Cells were treated with different doses of benidipine, mibefadil and nifedipine (0.1, 1.0, 5, 10, 20, and 50 μM) or vehicle, respectively, for 48 h. Supernatants were collected to detect aldosterone concentration by radioimmunoassay.

### Drug Treatment, Detection of Aldosterone and Cell Proliferation

The cells were treated with benidipine (10 μM), nifedipine (10 μM), mibefradil (10 μM), or vehicle, respectively, for 48 h. The supernatant in 24-well plates was harvested to detect aldosterone concentration by radioimmunoassay. The cells were taken for RNA assay. Cells cultured in 96-well plates were treated with benidipine (10 μM), nifedipine (10 μM), mibefradil (10 μM) or vehicle, respectively. Forty-eight hours later, the culture medium was changed to 100 μl fresh culture medium and 10 μl CCK-8, and cells were incubated for 2 h at 37°C in cell compartment. Absorbance was measured at 450 nm using a microplate reader.

At day 3 of primary culture, the medium was changed to low serum medium (DMEM/F12 medium supplemented with 2% FBS). APA cells were treated with ACTH (100 nM), ATII (100 nM), potassium (15 mM) and vehicle, with or without an addition of nifedipine, respectively, for 24 h. The supernatant collection, measurement of aldosterone concentration and detection of cell proliferation were conducted as aforementioned. Each group in proliferation experiments had 5 or 6 parallel wells, and each group in secretion experiments had 3 or 4 parallel wells.

### RNA Isolation and Real-Time PCR

Cells were lysed with Trizol reagent, and total RNA was isolated according to protocol provided by the manufacturer. Complementary DNA (cDNA) was synthesized from 1 μg of total RNA by using the PrimeScript™ RT reagent Kit with gDNA Eraser. PCR amplifications for aldosterone synthase (*CYP11B2*) gene were conducted by using TB Green™ Premix Ex Taq™ II on a LightCycler 480 PCR system. The relative gene expression levels were calculated by using the 2^(−ΔΔCT)^ method and were normalized against the expression of the human glyceraldehyde-3-phosphate dehydrogenase (GAPDH) gene. The primers used were from previous published studies (13). All real-time PCR was performed in triplicate for each sample.

### Amplification and Sequencing of *KCNJ5, ATP2B3, ATP1A1*, and *CACNA1D* Genes

Genomic DNA was isolated from fresh APA tissues or formalin-fixed paraffin-embedded (FFPE) tissues according to instructions. All the hot spot mutation regions of *KCNJ5, ATP2B3, ATP1A1*, and *CACNA1D* were amplified and sequenced on an ABI3730 DNA Analyzer (Applied Biosystems). The primers are shown in Table 2. GenBank accession number NM_000890 was used as a reference sequence for *KCNJ5* gene.

**Table 2:** The primers of *KCNJ5, ATP2B3, ATP1A1*, and *CACNA1D*.

**Primers**	**5^**′**^ to 3^**′**^**	**Tm (^**°**^C)**
ATP2B3-7F	GTGTCCATACCTCTTCTTCC	52
ATP2B3-7R	AGTTTATACTGCCACCAAGG	
ATP1A1-4F	TGGAGGAATTTGCTAGGTTT	52
ATP1A1-4R	AACGAAGGAAGAATGGATGA	
ATP1A1-8F	TGTCCTGCTACTGGAGAG	52
ATP1A1-8R	TAGGATAGCGGAAGAGTGTA	
ATP1A1-21F	TTCATCTGACCTCCAAGTT	52
ATP1A1-21R	GAAGCCTCAGGATTTGTTAG	
CACNA1D-6F	AAGGAGGCATGGTTAGGA	55
CACNA1D-6R	TTCTGAACATAGCTCACACT	
CACNA1D-8F	GAGCACTAACCTTCAGCAA	55
CACNA1D-8R	AACACGGAATCTCACAGAC	
CACNA1D-15F	GTCCTGCATGGGTGTTCTGA	55
CACNA1D-15R	ACGAAGTGCTTTTCGGGGAA	
CACNA1D-16F	CTGGCAATAGAGCGAGAC	55
CACNA1D-16R	AGATAACGACGGCAACAA	
CACNA1D-24F	CACGCTAACTGTGCAGGGA	55
CACNA1D-24R	TCAGCTCTGCCCAGAAGAG	
CACNA1D-28F	CCAATCTACAACCACCGCGT	55
CACNA1D-28R	GACCAAGGGACAGAAGCCAA	
CACNA1D-34F	ACGGTTCTTCCTCACTGTCG	55
CACNA1D-34R	CTTCAGCAGAGGCATTTGGCT	
KCNJ5-2aF	GAGGATTTCACGCCCTGAC	60
KCNJ5-2aR	CCCGGATATAAGCAATGAGC	
KCNJ5-2bF	CGCTTCAACTTGCTCGTCTT	60
KCNJ5-2bR	ATGAACTCCCCCTCTTTGGT	
KCNJ5-2cF	TCCAACAACGCAGTCATCTC	60
KCNJ5-2cR	CCAGTACCCCTCAAACCACA	
KCNJ5-3F	GATTGCATCATAATGCATGTAA	60
KCNJ5-3R	TTAGCCAGCACCTACAAGAG	

### Statistical Analysis

The data were expressed as mean ± SE or mean ± SD for normal distribution data. Differences between groups were analyzed with Student's *t*-test for the normal distribution. *P*-value < 0.05 was considered to be statistically significant. The statistical analyses were performed by employing the SPSS 21.0 software package.

## Results

### Dose-Effect of CCBs

**Figure 1:**
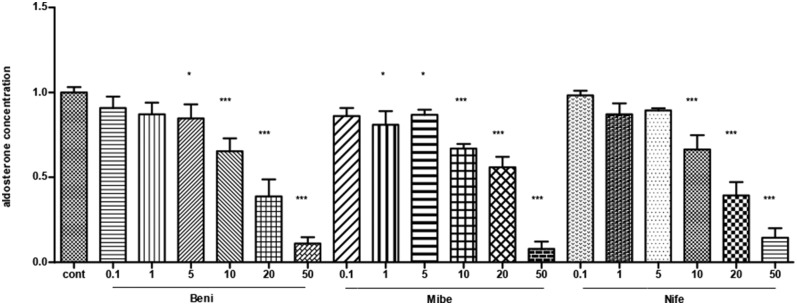
The dose-effect of CCBs on aldosterone secretion in APA cells (mean ± SE, *N* = 3). Cont, control; Beni, benidipine; Mibe, mibefradil; Nife, nifedipine; X axis means different concentration of Benidipine, mibefradil and nifedipine. The unit is μM. Aldosterone concentration in control group was set as 1. * *P* < 0.05; ***P < 0.001.

All the three CCBs dose-dependently inhibited basal aldosterone secretion with the minimum effective dose at 5, 1, 10 μM for benidipine, mibefadil and nifedipine, respectively (*P* < 0.05). We saw significant inhibition in all of the three groups at the concentration of 10 μM and at larger concentrations (Figure 1).

### Effects of Different CCBs on Aldosterone Secretion and Proliferation in APA Cells

**Figure 2:**
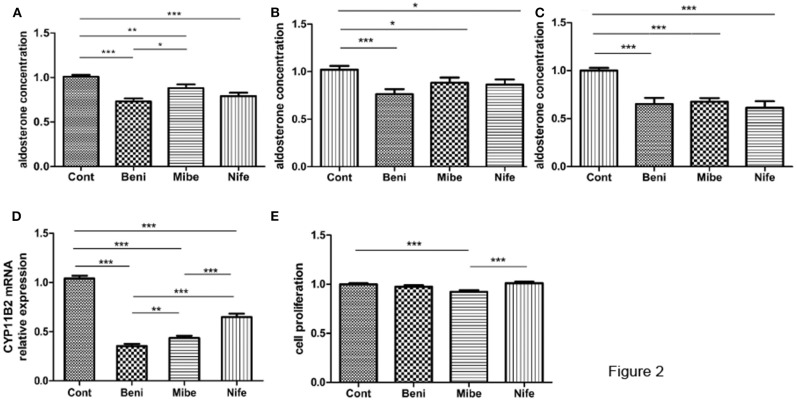
Effects of CCBs with a final concentration of 10 μM on aldosterone secretion and proliferation in APA cells. **(A)** Effects on aldosterone secretion (mean ± SE, *N* = 15); B and C. Effect on aldosterone secretion in different genotypes (mean ± SE) **(B)** *KCNJ5*^G151R^, *N* = 7; **(C)** *KCNJ5*^L168R^, *N* = 5; **(D)** Effects on CYP11B2 mRNA expression (mean ± SD, *N* = 9); **(E)** Effects on cell proliferation (mean ± SD, *N* = 3). Cont, control; Beni, benidipine; Mibe, mibefradil; Nife, nifedipine; Aldosterone concentration or cell proliferation in control group was set as 100%; *P < 0.05; **P < 0.01; ***P < 0.001.

Compared with the control (taken as 100%), aldosterone concentration was 75 ± 3%, 88 ± 4% and 79 ± 3% (mean ± SE) in benidipine, mibefradil and nifedipine group (each dose was 10 μM), respectively (Figure 2A). *P*-value was <0.0001, <0.005 and < 0.0001 respectively. Fourteen of 15 APAs included in this part of experiment had *KCNJ5* mutations, including amino acid changes p.G151R (*N* = 7), p.L168R (*N* = 5) and p.E145Q (*N* = 2). In both *KCNJ5*^G151R^ and *KCNJ5*^L168R^ APAs, all the three CCBs inhibited aldosterone secretion (Figures 2B,C). One of 15 APAs did not have any somatic gene mutation of *KCNJ5, ATP2B3, ATP1A1*, and *CACNA1D*.

APA cells from 9 out of 15 APAs were studied at mRNA level. All the three CCBs significantly inhibited CYP11B2 mRNA expression (Figure 2D). The inhibitory effect was strongest in benidipine group.

Mibefradil marginally inhibited cellular proliferation, while benidipine and nifedipine did not have such effect (Figure 2E). All the three APAs included in this study had *KCNJ5* gene mutation (two had p.G151R mutation and one had p.L168R mutation).

### Effects of ACTH, ATII and Potassium on Aldosterone Secretion and Proliferation in APA Cells

**Figure 3:**
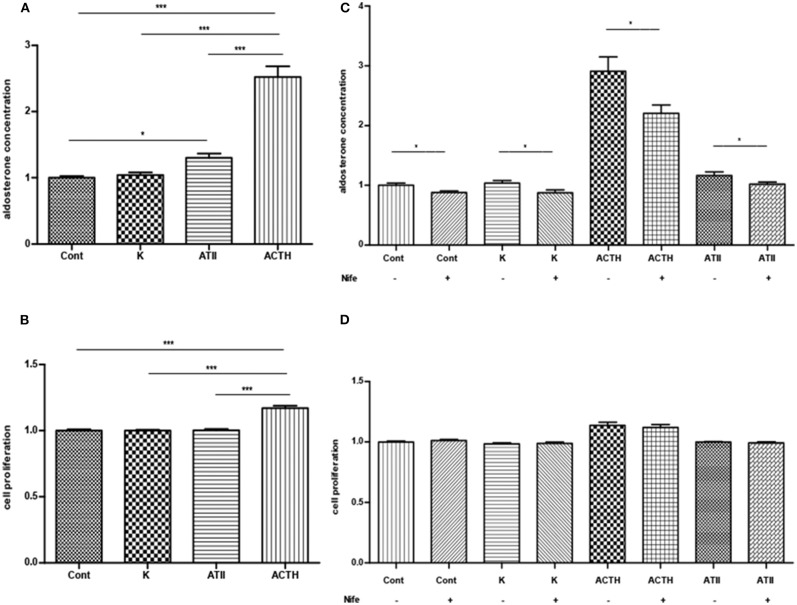
**(A,B)** Effects of ACTH, ATII and potassium on aldosterone secretion (**A**, mean ± SE, *N* = 7) and cell proliferation (**B**, mean ± SD, *N* = 6). **(C)** Effects of nifedipine on basal or ACTH-, ATII- and potassium-induced aldosterone secretion (mean ± SE, *N* = 4). **(D)** Effects of nifedipine on basal and ACTH-, ATII- and potassium-induced cell proliferation (mean ± SD, *N* = 4). Control, ACTH, ATII and potassium group were only compared with its corresponding group treated with nifedipine in **(C,D)**; Cont, control; K, potassium, a final concentration of 15 μM; ATII, angiotensin II, a final concentration of 100 nM; ACTH: a final concentration of 100 nM *P < 0.05; ***P < 0.001.

ACTH significantly stimulated aldosterone secretion and cell proliferation (Figures 3A,B). ATII significantly promoted the aldosterone secretion but exerted no effect on cell proliferation. The stimulatory effect of ACTH on aldosterone secretion was much stronger than that of ATII. Potassium did not affect aldosterone secretion or cell proliferation. All APAs included in this part of experiment had *KCNJ5* mutation, including p.G151R, p.L168R and p.I157_E159del.

### Effects of CCBs on Aldosterone Secretion and Proliferation in APA Cells Treated With ACTH, ATII and Potassium

Nifedipine inhibited the basal and ACTH-, ATII-elicited aldosterone secretion. The inhibition rate in each group was 12 ± 3%, 22 ± 3% and 11 ± 4% respectively (mean ± SE). Nifedipine inhibited 15 ± 4% of aldosterone secretion in the potassium group (Figure 3C). Nifedipine did not change the cell proliferation in each group (Figure 3D). All APAs included in this study had *KCNJ5* mutation, including p.G151R and p.L168R.

## Discussion

Both L-type VGCC and T-type VGCC are expressed in human ZAG and APA cells (14). L-type VGCC activates at −50 mV, peaks at 0 mV and inactivates slowly. T-type VGCC is a low-voltage-activated and transient calcium channel, which activates at −65 mV and inactivates quickly (15). L-type or T-type VGCC opening triggers calcium influx, and thereby increases intracellular calcium concentration, which is the main mechanism by which aldosterone secretion is promoted in gene- mutated APA. Specific CCBs targeting different VGCCs might have different effect on APA cells.

Our study, by using primary cultured APA cells, demonstrated that CCBs inhibit aldosterone secretion. CYP11B2 is the terminal enzyme and the most important rate-limiting enzyme in the production of aldosterone. CYP11B2 mRNA expressions were also inhibited by CCBs in the primary culture of APA cells. As compared to mibefradil and nifedipine, benidipine showed a stronger inhibitory effect on CYP11B2 mRNA expression, suggesting that L/T-type CCBs might be more potent than L-type CCB or T-type CCB alone. Since T-type CCB are not approved to use in patients (16), L/T-type CCB was thought to be more promising than L-type CCB in the treatment of APA patients.

Twenty three of 24 APAs enrolled in the study had *KCNJ5* gene mutation. No *ATP2B3, ATP1A1*, and *CACNA1D* mutation were detected. T-type VGCC and L-type VGCC are activated in the condition of mutated *CACNA1H* and *CACNA1D* respectively. We postulated that CCBs might have a similar or better inhibitory effect in cases with *CACNA1H* and *CACNA1D* mutated adrenal cells. However, for us it is hard to verify this hypothesis since patients with *CACNA1H* or *CACNA1D* mutated APAs are rare. In our hands, most patients with APA had *KCNJ5* mutation and all of them corresponding hypokalemia. We reported a high frequency of *KCNJ5* mutation in Chinese APAs. It suggested that *KCNJ5* mutations were more frequent in APAs with hypokalemia since all the APA patients in our study had hypokalemia.

ACTH, ATII and potassium are the main aldosterone secretagogue in normal ZAG. In this study, we found that in APA cells, ACTH and ATII significantly stimulate aldosterone secretion, and ACTH was more effective than ATII, which is somewhat inconsistent with the fact that ATII is thought to be the major regulator in ZAG (17, 18). A previous clinical observation supports that APA patients respond better to ACTH than to ATII in promoting aldosterone secretion (19). In this study, however, ACTH and ATII infusion could have also stimulated the normal adrenal gland in addition to the adenoma invalidating the results and conclusions. Surprisingly, we found that potassium, even at high dose such as 15 mM, could not stimulate aldosterone secretion, which is different from the results obtained in ZAG cells (20). We speculate that in APAs with *KCNJ5* mutations chronic depolarization of cell membrane occurs, elevating extracellular potassium levels could changing the membrane potential causing an increase in aldosterone production. Our study shows that nifedipine inhibits aldosterone secretion at base-line, and in ACTH- and ATII-stimulated conditions. In normal ZAG cells, ACTH functions by binding to ACTH receptors on the cell surface stimulating adenylyl cyclase and thereby causing an increase in intracellular cAMP and subsequent activation of protein kinase A. However, until now the precise mechanisms are not fully understood. ATII activates Janus Kinase-2/insulin receptor substrate 1- insulin receptor substrate 2/phosphatidylinositol3-kinase/c-Jun N-terminal kinase/extracellular regulated protein kinases via Ang II receptor type 1 (21). Besides, ATII can also bring about depolarization (22) of the ZAG cell membranes opening VGCC and leading to an increased intracellular calcium concentration (5). Our result suggests that in APA, the stimulatory action of ACTH and ATII on aldosterone secretion might be partially mediated via VGCC.

## Conclusions

In this study, we examined the effects of CCBs on primary cell cultures of APA. We found that nifedipine, mibefradil and benidipine significantly inhibit aldosterone secretion.

## Data Availability Statement

The datasets generated for this study are available on request to the corresponding author.

## Ethics Statement

Informed consent was obtained from all subjects and the studies were approved by the PUMCH's Ethics Committee for Human Research with the IRB approval numbers being JS-1595 and JS-1596. The patients/participants provided their written informed consent to participate in this study.

## Author Contributions

FW performed the experiments and analyzed the data. XM and FW collected the clinical data. YZ and JW collected tumor tissues. AT instruct the whole study. AT and YL edited and revised the manuscript. All authors approved the final version of manuscript.

## Conflict of Interest

The authors declare that the research was conducted in the absence of any commercial or financial relationships that could be construed as a potential conflict of interest.
